# Editorial: Molecular Pathology of HTLV-1

**DOI:** 10.3389/fmicb.2018.03069

**Published:** 2018-12-12

**Authors:** Umberto Bertazzoni, Vincenzo Ciminale, Maria Grazia Romanelli

**Affiliations:** ^1^Department of Neurosciences, Biomedicine and Movement Sciences, Section of Biology and Genetics, University of Verona, Verona, Italy; ^2^Department of Surgery, Oncology, and Gastroenterology, University of Padua, Padua, Italy; ^3^Veneto Institute of Oncology IOV – IRCCS, Padua, Italy

**Keywords:** HTLV-1, TAX 1, HBZ, ATL, HAM/TSP

Human T-cell leukemia virus type 1 (HTLV-1) was the first human retrovirus discovered. It is estimated that 10–20 million people are infected worldwide by this oncogenic virus. About 5–10% of HTLV-1-infected individuals are at risk of developing either a fatal malignancy, adult T-cell leukemia (ATL), or a chronic progressive neuroinflammatory disease, called HTLV-associated myelopathy/tropical spastic paraparesis (HAM/TSP). Both diseases are incurable at present.

Many issues concerning HTLV-1's life cycle and pathobiology are still unsolved or controversial, and new approaches for prognostic stratification of patients and eradication of HTLV-1 infection are in high demand (Willems et al., 2017).

The present Research Topic is focused on the following themes:

1. Functional analyses and oncogenic potential of HTLV-1 regulatory proteins.
2. HTLV-1-associated diseases and new therapeutic developments.

## Functional Analyses and Oncogenic Potential of HTLV-1 Regulatory Proteins

Baratella et al. discuss the possible role of HBZ in the progression toward HTLV-1-associated diseases. One key finding of these authors is the exclusive cytoplasmic localization of HBZ in cells from HAM/TSP patients, which is in striking contrast to its nuclear localization in ATL cells. This finding suggests a correlation between HBZ's intracellular compartmentalization and the clinical outcome of HTLV-1 infection. The authors also propose that the cytoplasmic localization of HBZ in PBMC of HAM/TSP patients could be exploited as a molecular marker of disease. Kulkarni and Bangham Kulkarni and Bangham discuss the interplay between Tax and HBZ as well the influence of micro-environmental changes on latency and reactivation kinetics of the provirus. The authors present a comprehensive summary of factors that induce and inhibit plus-and minus-strand transcription of the provirus and propose that the binding of the cellular factor CTCF to the provirus produces abnormal chromatin loops, resulting in changes of cellular gene expression and possibly favoring the development of ATL. A research article by Gazon et al. sheds light on the long-standing hypothesis that Tax must be expressed in cycles to allow cell survival (Yoshida, 1987). By analyzing viral expression in HTLV-1 infected individuals and in sheep infected with bovine leukemia virus (BLV, a retrovirus closely related to HTLV-1 that produces B-cell neoplasias), the authors provide evidence for alternating expression of the plus- and minus proviral strands. They describe a model based on transient bursts of Tax-driven sense-strand transcription, followed by antisense RNA synthesis and silencing of plus strand synthesis. This mechanism would allow cell proliferation in the presence of a strong host immune response against Tax. Tanaka and Matsuoka review the cellular targets of HTLV infection *in vivo* focusing on the evidence indicating that HTLV-1 infects hematopoietic stem cells (HSC) *in vivo* and that HBZ influences early stages of hematopoietic cell differentiation and induces infected progenitor cells to abnormally differentiate into Treg-like T-cells. The authors also highlight the ability of HBZ to affect cell turnover by inducing genomic instability through upregulation of miR-17 and miR-21, which inhibit the expression of OBFC2A, a single-stranded DNA-binding protein that protects genome stability. Using affinity-tagged protein and shotgun proteomics, Panfil et al. identified the UBR5 E3 ubiquitin ligase as a novel binding partner of HBZ. The authors show that this interaction results in HBZ ubiquitination. Interestingly, UBR5 was found to be overexpressed in T leukemia/lymphoma cell lines and during the later stage of T-cell transformation *in vitro*, while loss of UBR5 decreased cellular proliferation, suggesting a role for UBR5 in maintaining the proliferative phenotype of transformed T-cells. Gazon et al. discuss the effects of Tax and HBZ on the transcription factors AP-1, ATF/CREB, and Maf and the contribution of these pathways to HTLV-1-induced cellular transformation. The authors dissect the intricate mechanisms of AP-1 pathway activation and propose that AP-1 is activated in HTLV-1-infected T-cells through Tax-dependent and Tax-independent mechanisms. Experimental evidence suggests that HBZ interacts with all three members of the Jun family: while sequestering c-Jun and JunB in HBZ-containing nuclear bodies, it cooperates with JunD to enhance transcription of the 3′LTR and hTERT. Several aspects of NF-κB pathway deregulation mediated by Tax and HBZ are reviewed by Fochi et al. By focusing on differences in the abilities of Tax-1/HBZ and their HTLV-2 homologs Tax-2/APH-2 to interact with host factors, the authors summarize the distinct impacts of Tax and HBZ on NF-κB deregulation and their effects in cell survival and proliferation. In an investigation of the influence of the microRNA network on the turnover of HTLV-1 infected cells, Sharma et al. provide evidence that HTLV-1 infection induces upregulation of miR-34a. Based on their findings, the authors propose that high miR-34a levels may provide a selective advantage to HTLV-1 infected cells *in vivo* that persists during the transformation process. Recent studies have shown that the p8 accessory protein of HTLV-1 induces T-cell conjugates and cellular conduits that may facilitate virus transmission. Donhauser et al. have developed a novel method based on flow cytometry for the automated quantitation of p8 transfer between cells. Using this method in time course experiments the authors show that p8 is rapidly transferred between Jurkat T-cells and that actin polymerization affects this process. Cavallari et al. present a review of the interactions between mitochondria and proteins coded by HTLV-1 and the human tumor viruses, Epstein-Barr virus (EBV), Kaposi's sarcoma-associated herpesvirus (KSHV), hepatitis viruses B and C (HBV and HCV), and human papillomavirus (HPV). The authors discuss how these interactions may contribute to viral replication, persistence and transformation and how these functions are connected to the crucial role of mitochondria in cellular bioenergetics, apoptosis, innate immunity, and redox balance.

## Controlling HTLV-1-Associated Diseases

Tagaya and Gallo provide an insightful comparison of the oncogenicity of HTLV-1, other human oncoviruses and *Helicobacter pylori*. Interestingly, the lifetime risk to develop a malignancy is markedly higher in patients infected with HTLV-1—compared to EBV, KSHV, HBV, HCV, HPV, and *H. pylori*. Furthermore, the oncogenic mechanism of HTLV-1 is more direct. These striking features of HTLV-1 are often overlooked, possibly because of the low prevalence of the virus in the US and Europe. Based on these considerations the authors affirm the notion that this most carcinogenic virus deserves the “leukemia” name. Based its high oncogenic potential, Gallo et al. thus recommend to return to the virus' original name, “Human T-cell leukemia virus” in place of the more generic “Human T-cell lymphotropic virus.”

Pasquier et al. discuss the difficulties in treating HTLV-1 patients and the current therapies available, in particular for ATL, and provide a schematic representation of the two modes of HTLV-1 transmission, i.e., through *de novo* by virus particles and clonal expansion of cells harboring the provirus. Using a novel *in vitro* assay, the authors screened an impressive number of drugs, and showed for the first time that the acyclic nucleoside phosphonates Adefovir dipivoxil and Tenofovir disporoxil are much more effective at blocking HTLV-1 transmission compared to the conventional nucleoside reverse transcriptase inhibitor AZT. In their review, Marino-Merlo et al. provide an up-to-date overview of recent efforts to understand the mechanisms involved in current therapeutic regimens for ATL. Targeted biological therapies for ATL are thoroughly described in the prospective to find novel molecular targets in ATL therapy. Based on the accumulated evidence, the authors point out that the control of virus spread is a crucial aspect in ATL therapy.

Yamagishi et al. provide an overview of the multiple genetic and epigenetic aberrations that characterize *in vivo* HTLV-1 infection in its initial stages, when both Tax and HBZ are at work. The authors highlight the crucial, and apparent transient role of Tax in shaping the host cell genome and epigenome with the active, persistent role of HBZ, which further potentiates initiation and progression to ATL and HTLV-1 associated diseases. Queiroz et al. investigated the inflammatory response in HTLV-1 associated diseases and analyzed the relation between the IFNG+874A/T polymorphism and the progression of HTLV-1 infection to symptomatic disease. Patients carrying the polymorphic allele showed significantly higher levels of IFN-gamma than those carrying the wild-type allele, suggesting that asymptomatic carriers with the T allele of IFNG+874A/T and a high proviral load have a high probability of developing HTLV-1 associated inflammatory diseases. Moodad et al. provide a comprehensive review of the mouse models of ATL and HTLV-I infection. Transgenic animals for Tax and HBZ, as well as knock-outs for key cellular regulators provide useful models to understand the role of viral and host genes in the development of ATL. In addition, models based on xenografts and humanized immune-deficient mice provide a valuable platform to test new therapies against ATL.

An interesting new murine preclinical ATL model is presented in the original paper by Vicario et al.. The authors describe experiments in which they injected an HTLV-1-immortalized T-cell line either alone or together with normal primary fibroblasts into NGS mice, and demonstrate that fibroblasts strongly enhance HTLV-1 the growth of this cell line, which partly recapitulates the *in vivo* pattern of the lymphoma variant of ATL. This model may be useful to test novel therapeutic interventions for the aggressive lymphoma subtype of ATL.

Enose-Akahata et al. review the role of Tax and HBZ in the pathogenesis of HAM/TSP. They describe the infiltration of HTLV-1-infected lymphocytes into the CNS and their chronic release of Tax, which is capable of producing a neurotoxic effect on the long axons of corticospinal tracts involved in progressive neurological disease. It appears that HBZ expression is able to confer a pro-inflammatory phenotype to infected T-cells, a property that would position HBZ as an important factor in the HAM/TSP disease process.

Sato et al. describe an analysis of 453 HAM/TSP patients enrolled in the Japanese “AAM-net” registry. By measuring eight candidate biomarkers in PBMCs, serum and cerebrospinal fluid (CSF) samples, they demonstrate that the levels of neopterin and CXCL10 in CSF represent valuable markers to discriminate among rapid, slow and very slow progressors. Based on these results, the authors propose new classification criteria for disease activity which will aid in optimizing treatment.

Using an integrated *ex vivo* approach, Leal et al. show that antiretroviral genes and HAM/TSP cluster in two distinct “proviral/antiviral” classes, of which the TRIM5a/TRIM22/BST2 antiviral subset is selectively up-regulated by IFN-beta signaling in HAM/TSP. These findings provide mechanistic evidence for the *in vivo* immunovirological effect of IFN-β treatment in HAM/TSP and identify novel biomarkers as well as possible therapeutic targets.

## Conluding Remarks

Nearly 40 years of research have yielded important insights into diverse aspects of the biology of HTLV-1 infection and pathogenesis. Nevertheless, we are still quite far from deciphering the complexity of its associated diseases. We hope that the broad range of issues addressed in this Research Topic will provide new perspectives and groundwork for future studies aimed at unraveling HTLV-1 pathogenesis and lead to the development of novel tools to eradicate the virus and its diseases.

## Author Contributions

All authors UB, VC, and MGR contributed equally to the writing of the manuscript.

### Conflict of Interest Statement

The authors declare that the research was conducted in the absence of any commercial or financial relationships that could be construed as a potential conflict of interest.
